# Tumor pH-Responsive Nanocarriers With Light-Activatable Drug Release for Chemo-Photodynamic Therapy of Breast Cancer

**DOI:** 10.3389/fchem.2022.905645

**Published:** 2022-06-22

**Authors:** Zhang Zhang, An Gao, Chunyang Sun

**Affiliations:** Department of Radiology and Tianjin Key Laboratory of Functional Imaging, Tianjin Medical University General Hospital, Tianjin, China

**Keywords:** tumor acidity responsive, nanocarrier, TAT presenting, on-demand drug release, chemo-photodynamic combination therapy

## Abstract

Developing bioresponsive nanocarriers with particular tumor cell targeting and on-demand payload release has remained a great challenge for combined chemo-photodynamic therapy (chemo-PDT). In this study, an intelligent nanocarrier (^DA^TAT-NP_Ce6_) responded to hierarchical endogenous tumor pH, and an exogenous red light was developed through a simple mixed micelle approach. The outside TAT ligand was masked to prevent an unexpected interaction in blood circulation. Following the accumulation of ^DA^TAT-NP_Ce6_ in tumor tissues, tumor acidity at pH ∼6.5 recovered its targeting ability *via* triggering DA moiety degradation. Furthermore, the cascaded chemo-PDT was accomplished through light-stimulated nanocarrier disassembly and doxorubicin (DOX) release. Taking advantage of stability and controllability, this work provides a facile approach to designing bioresponsive nanocarriers and represents a proof-of-concept combinatorial chemo-PDT treatment.

## Introduction

Chemotherapy is dominantly used alone or with surgery or radiation therapy for cancer treatment (Fridman et al., 2017; Harbeck and Gnant, 2017; Waks and Winer, 2019). However, its therapeutic outcomes are always unsatisfactory because of insufficient blood concentration, lack of specificity, and severe side effects (Staff et al., 2017; Oun et al., 2018). Photodynamic therapy (PDT), which employs photosensitizers (PSs) to generate abundant reactive oxygen species (ROS) for cell killing, has emerged as an alternative treatment modality against malignant tumors (Lam et al., 2001; Buytaert et al., 2007; Zhou et al., 2016; Ni et al., 2018). Moreover, the combination of chemotherapy and PDT is considered to rationally improve therapeutic efficiency through complementary molecular mechanisms. In recent years, desirable nanocarriers have been proposed to specifically and effectively co-deliver chemotherapeutic agents and PSs to tumoral cells (Luo et al., 2018; Purushothaman et al., 2019; Jin et al., 2020). Among various nanocarriers, the ROS-sensitive nanocarrier is an attractive candidate because it can ensure PDT and chemotherapy work cooperatively rather than separately (Dai et al., 2016; Cao et al., 2018; Wei et al., 2018; Zhang et al., 2020; Zeng et al., 2021). For instance, He et al. reported a ROS-responsive nanosized micelle formed by polymer-conjugated doxorubicin (Wang et al., 2019). Under laser irradiation in designated time and space, this nanoplatform produced ROS, further triggering the disassembly of nanocarriers to achieve precise on-demand cargo release for improving chemo-photodynamic therapy and reducing off-target toxicity.

Except for intracellular proper functioning, both efficient cellular internalization and tumor accumulation are necessities for ROS-responsive nanocarrier design (Liu et al., 2019; Raj et al., 2021; Yang et al., 2021). Despite great effort, decorating nanocarriers with cell-penetrating peptides (CPPs, e.g., TAT or R9) is limited by unavoidable blood clearance and non-selective toxicity *in vivo* (Sarko et al., 2010; Bolhassani et al., 2017; Habault and Poyet, 2019). Therefore, achieving precise CPP presentation, shielding in blood and exposure to the desired site of action (i.e., tumors), is essential. Unlike other cells in normal organs, tumoral cells utilize energy that comes from oxygen-independent glycolysis (known as the Warburg effect) (Vander Heiden et al., 2009; Vaupel and Multhoff, 2021). As a result, the excess lactate and CO_2_ efflux by tumoral cells acidify the extracellular tumor matrix to pH ∼6.5–6.8 (Du et al., 2018; Klaus and Deshmukh, 2021). The significantly lower pH microenvironment inspires us to propose bioresponsive nanocarriers with CPP modification to maximize their delivery efficacy to tumor tissues. Fortunately, growing evidence has also indicated that dimethyl maleate (DA) moiety was extraordinarily sensitive to extracellular acidity (Kirby and Lancaster 1972; Du et al., 2011; Lee et al., 2011; Wang et al., 2013; Kang et al., 2014).

**FIGURE 1:**
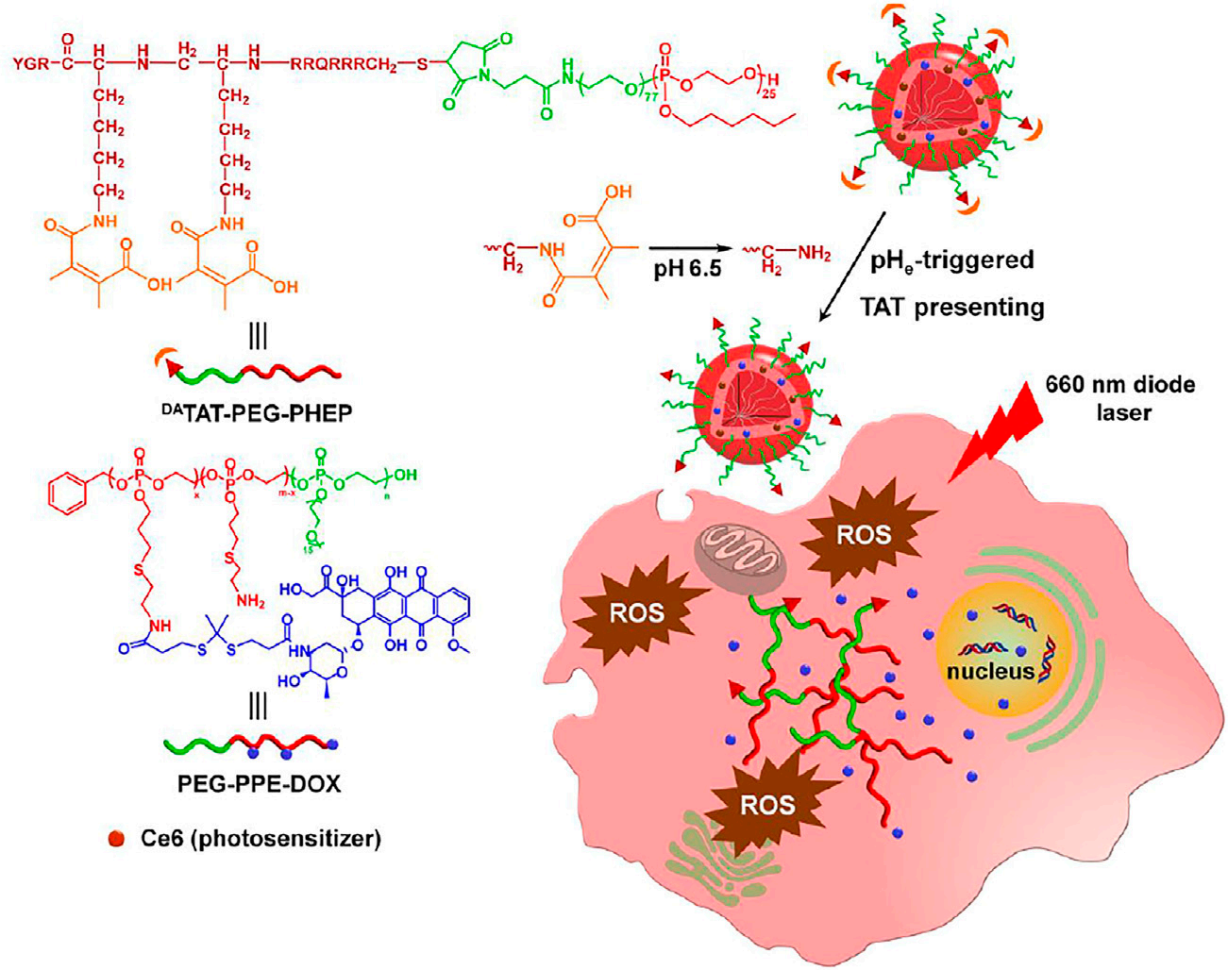
Scheme of tumor-pH-triggered TAT presenting drug delivery and light-activated DOX release for combined chemo-PDT.

In light of these findings, we developed a bioresponsive system capable of TAT deshielding at acidity and light-activable drug release for combined chemo-PDT. The designed nanocarriers (^DA^TAT-NP_Ce6_) were self-assembled from TAT-modified poly(ethylene glycol)-polyphosphoesters (TAT-PEG-PHEP), doxorubicin (DOX) conjugated copolymers containing ROS-responsive thioketal linkers (PEG-PPE-DOX), and photosensitizer chlorin e6 (Ce6, Figure 1). The TAT function of ^DA^TAT-NP_Ce6_ was shielded by DA moiety after intravenous injection. Following extravasation into the tumor matrix *via* the EPR effect, localized lower pH could degrade the DA group to reactivate TAT function to raise tumoral cell uptake and accumulation of ^DA^TAT-NP_Ce6_. Next, 660 nm light radiation to Ce6 produced sufficient ROS to not only perform cell killing but also lead to thioketal cleavage for cytoplasmic release of DOX payload. Thus, ^DA^TAT-NP_Ce6_ cascade-amplified chemo-PDT effects and its effectiveness plus light radiation were examined in both *in vitro* and *in vivo* studies.

## Materials and Methods

### Materials

Maleimide groups functional poly(ethylene glycol) (Mal-PEG, Mw = 3,400) was purchased from Shanghai ToYongBio Tech. Inc (China). The TAT peptide was supplied from Chinese Peptide Company, Ltd. TAT-modified PEGylated polyphosphoesters (TAT-PEG-PHEP) and DOX conjugated polyphosphoesters (PEG-PPE-DOX) were synthesized according to previous reports (Li et al., 2017; Li et al., 2018). 3-(4,5-Dimethylthiazol-2-yl)-2,5-diphenyl tetrazolium bromide (MTT) was purchased from Sigma-Aldrich Chemical Co., Ltd. Dulbecco’s modified Eagle’s medium (DMEM) and fetal bovine serum (FBS) were purchased from Life Technologies Corporation (Gibco, United States). All other reagents were purchased from Shanghai Aladdin Bio-Chem Technology Co., Ltd. and used as received.

### Preparation of Ce6-Loaded Nanoparticles

TAT-PEG-PHEP of 4 mg, PEG-PPE-DOX of 16 mg, and Ce6 (2.0 mg) were dissolved in 2.0 ml of DMF, and then slowly added to 20 ml of ddH_2_O. After stirring for 30 min, the organic solvent and free small molecule drug were removed by dialysis against ddH_2_O and subsequent centrifugation (1,000 g, 10 min). The nanoparticles were denoted by TAT-NP_Ce6_. To prepare nanocarriers with pH sensitivity, five equivalents (relative to amines of TAT) of 2,3-dimethylmaleic anhydride were gradually mixed with TAT-NP_Ce6_ at pH 8-9. Following reaction at room temperature for 4 h, the unreacted 2,3-dimethylmaleic anhydride was removed by ultrafiltration (MWCO 3400 Da). The obtained nanocarriers were denoted ^DA^TAT-NP_Ce6_. On the other hand, the tumor pH-insensitive ^SA^TAT-NP_Ce6_ was synthesized using succinic anhydride.

### Cellular Uptake of pH-Responsive Nanocarriers

MDA-MB-231 cells in 24-well plates were incubated with TAT-NP_Ce6_, ^SA^TAT-NP_Ce6_, or ^DA^TAT-NP_Ce6_ which was pretreated at either pH 7.4 or 6.5, washed, and harvested. The internalized DOX was then detected by flow cytometry (BD FACS Calibur). The Ce6 content in MDA-MB-231 cells was quantitatively measured by HPLC after the cell lysis.

For the confocal laser scanning microscope (CLSM) observation, MDA-MB-231 cells were seeded and incubated with TAT-NP_Ce6_, ^SA^TAT-NP_Ce6,_ or ^DA^TAT-NP_Ce6_ which was pretreated at either pH 7.4 or 6.5. Following 4 h of incubation, the cells were washed and fixed with 4% paraformaldehyde, then stained with Alexa Fluor^®^ 488 phalloidin and DAPI sequentially following the standard protocol. The cells were then imaged on a Zeiss LSM 810 microscope.

### Cell Killing of ^DA^TAT-NP_Ce6_ *In Vitro*

To determine the cytotoxicity of nanocarriers without drug loading, MDA-MB-231 cells were seeded in a 96-well plate (10,000 cells per well) and incubated with TAT-NP, ^SA^TAT-NP, or ^DA^TAT-NP for 72 h. To determine the efficacy of combined chemo-PDT therapy, MDA-MB-231 cells were seeded in a 96-well plate (10,000 cells per well). TAT-NP_Ce6_, ^SA^TAT-NP_Ce6_, or ^DA^TAT-NP_Ce6_ was pretreated in phosphate buffer at either pH 7.4 or 6.5. After the treatment for 2 h, the nanocarriers were diluted by DMEM medium. The MDA-MB-231 cells were then incubated with the DMEM medium containing nanocarriers at different DOX concentrations for 12 h. Following the replacement of the DMEM medium without nanocarriers, the cells were irradiated by a 660 nm laser for 10 min at a power density of 100 mW/cm^2^. After further incubation for 60 h, the cell viabilities were measured by a standard MTT assay.

### Pharmacokinetic and Biodistribution of ^DA^TAT-NP_Ce6_

BALB/c mice (female) were randomly divided into four groups (*n* = 4). The mice were administrated with free Ce6, TAT-NP_Ce6_, ^SA^TAT-NP_Ce6_, or ^DA^TAT-NP_Ce6_ through *i. v.* injection. The equivalent dose of Ce6 was 10 mg per kg body weight. At 0.167, 0.5, 1, 2, 4, 8, 12, 24, 48, and 72 h post-injection, blood samples were collected from the retroorbital plexus. After the centrifugation, the DOX concentration in plasma was measured using HPLC.

To determine the biodistribution of ^DA^TAT-NP_Ce6_ *in vivo*, BALB/c nude mice bearing MDA-MB-231 xenografts received a systemic injection of free Ce6, TAT-NP_Ce6_, ^SA^TAT-NP_Ce6_, or ^DA^TAT-NP_Ce6_ (*n* = 4). The equivalent dose of Ce6 was 10 mg per kg body weight. At 12, 24, and 48 h post-injection, the major organs and tumor tissues were harvested, and the DOX content was quantitatively detected by HPLC.

### Antitumor Effect *In Vivo*

MDA-MB-231 tumor-bearing BALB/c nude mice (female) were randomly divided into five groups (*n* = 6). The date was recorded as day 0 when the tumor volume was about 100 mm^3^. On days 0, 7, and 14, the mice were administrated with PBS, free Ce6&DOX, TAT-NP_Ce6_, ^SA^TAT-NP_Ce6_, or ^DA^TAT-NP_Ce6_ through tail vein ([DOX] = 5 mg/kg body weight). At 24 h post-injection, the tumor tissue was irradiated with a 660 nm laser for 10 min at a power density of 200 mW/cm^2^. The tumor growth and mice's body weight were recorded every 2 days. The tumor volume was calculated as 0.5 × length × width^2^. After the sacrifice on day 19, the blood sample was collected for ELISA examination.

## Results and Discussions

### Synthesis and Characterization of TAT-Masked Nanocarriers

**FIGURE 2:**
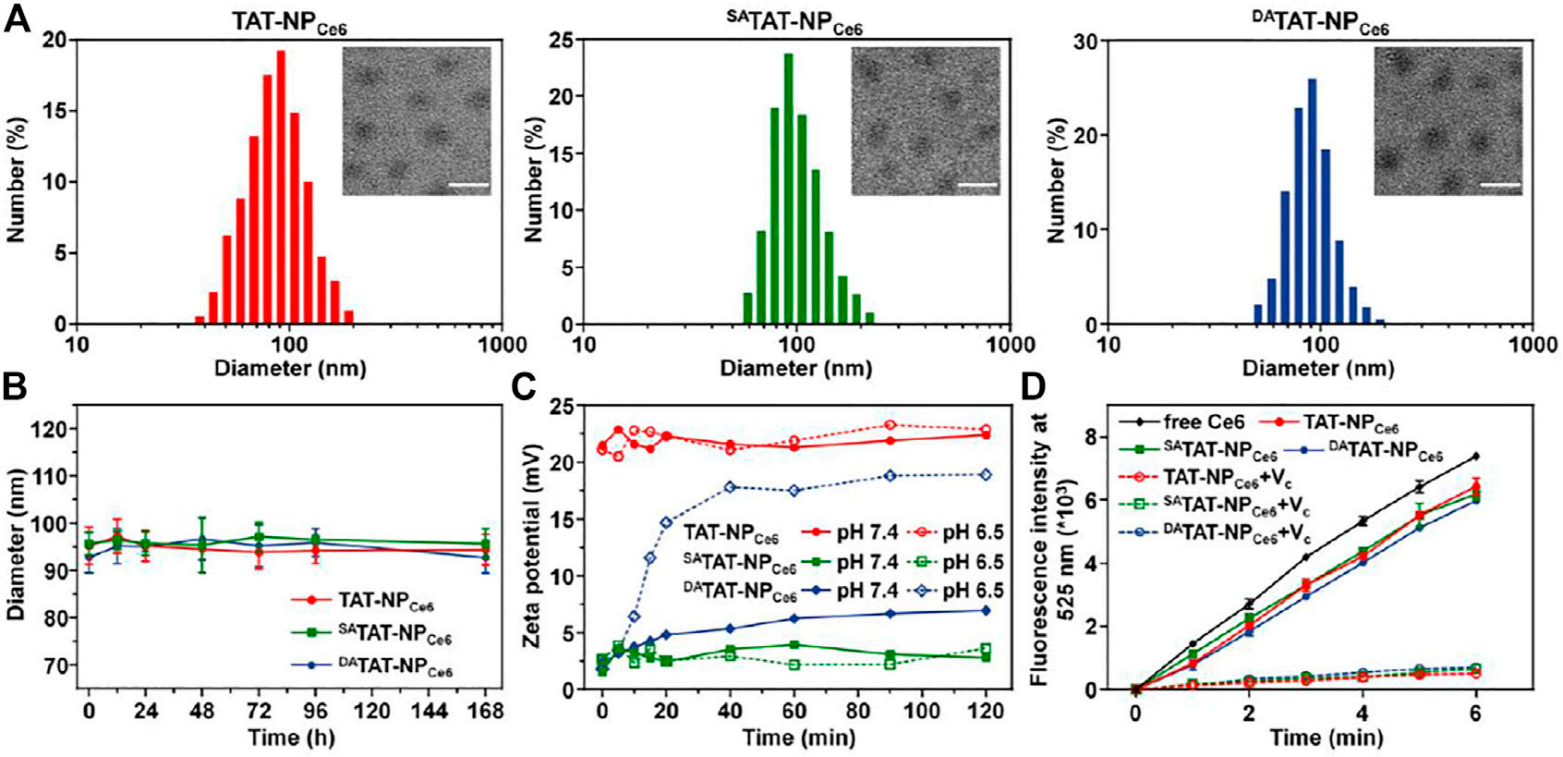
**(A)** Size distribution and morphology of TAT-NP_Ce6_, ^SA^TAT-NP_Ce6_, and ^DA^TAT-NP_Ce6_. The scale bar is 100 μm. **(B)** Diameter change in PBS solution. **(C)** Zeta potential change of TAT-NP_Ce6_, ^SA^TAT-NP_Ce6_, and ^DA^TAT-NP_Ce6_ at different pH conditions. **(D)** DCF fluorescence intensity (E_m_ = 525 nm) in various groups. Vitamin C (V_C_) was used as an ROS scavenger.

Considering the synthetic difficulty in simultaneous modification of TAT peptide and DOX on a single copolymer, the pH_e_-responsive nanocarriers with light-activated disassembly were designed to be a mixed micellar formulation. TAT-modified PEGylated polyphosphoesters (TAT-PEG-PHEP) and DOX conjugated polyphosphoesters (PEG-PPE-DOX) were synthesized according to previous reports. Thereafter, hydrophobic Ce6 (both amphiphilic TAT-PEG-PHEP and PEG-PPE-DOX were self-assembled into mixed micellae), and the obtained nanoparticles were denoted by TAT-NP_Ce6_. To integrate pH_e_-responsive TAT presenting, 2,3-dimethylmaleic anhydride was used to react with the lysine residue amines of TAT-NP_Ce6_, and the obtained nanocarriers were denoted by ^DA^TAT-NP_Ce6_. Meanwhile, the control nanocarrier without pH sensitivity (^SA^TAT-NP_Ce6_) was prepared *via* a similar route, whereas the 2,3-dimethylmaleic anhydride was replaced by succinic anhydride. As shown in Figure 2A, dynamic light scattering (DLS) measurement demonstrated that the average diameter of resultant TAT-NP_Ce6_, ^SA^TAT-NP_Ce6_, and ^DA^TAT-NP_Ce6_ was around 90 nm (polydispersity index <0.2). The TEM images illustrated that TAT-NP_Ce6_, ^SA^TAT-NP_Ce6_, and ^DA^TAT-NP_Ce6_ maintained a circular morphology with a slightly smaller size than that of DLS results. The DOX and Ce6 loading contents of these nanocarriers were ca. 17.42 and 2.81%, respectively. Owing to the protection and stabilization of the outer PEG shell, all three nanocarriers maintained their original size even after incubating in phosphate buffer (pH 7.4) for 7 days (Figure 2B).

DA moieties containing carboxyl would be selectively degraded at pH 6.5 to expose the original amine groups of the lysine residues for TAT function regeneration (Jin et al., 2013). Thereafter, we monitored the zeta potential changes of TAT-NP_Ce6_, ^SA^TAT-NP_Ce6_, and ^DA^TAT-NP_Ce6_ at either pH 7.4 or 6.5. As shown in Figure 2C, the zeta potentials of both TAT-NP_Ce6_ and ^SA^TAT-NP_Ce6_ maintained roughly unchanged regardless of pH conditions. Contrary to slight elevation at pH 7.4, the zeta potential of ^DA^TAT-NP_Ce6_ at pH 6.5 gradually increased from 1.8 to 18.9 mV, which is comparable to that of TAT-NP_Ce6_, indicating the accelerated degradation pattern of DA moieties modified on TAT peptide. Furthermore, the amine groups left by DA degradation were measured using a fluorescamine sensor. As shown in Supplementary Figure S1, the degradation of DA of ^DA^TAT-NP at pH 6.5 (88.52%) was significantly higher than that at pH 7.4 (25.96%), while ∼90% of SA moieties of ^SA^TAT-NP remained at either pH 7.4 or 6.5.

Next, the ROS generation of Ce6-loaded nanoparticles under laser irradiation was detected using 2′,7′-dichlorofluorescin diacetate (DCF-DA) as a ROS probe. As displayed in Figure 2D, TAT-NP_Ce6_, ^SA^TAT-NP_Ce6_, and ^DA^TAT-NP_Ce6_ showed a remarkable DCF fluorescence growth upon 660 nm laser exposure. In comparison with free Ce6, the decreased fluorescence intensity of Ce6-loaded nanoparticles may be attributed to the quenchable singlet oxygen quantum yields after Ce6 encapsulation. It was worth noting that vitamin C (ROS scavenger) could significantly inhibit the ROS production of Ce6-loaded nanoparticles, indicating the produced ROS is produced by Ce6 during the PDT process (Shivaprasad et al., 2021).

### Light-Activated Disassembly and Drug Release From ^DA^TAT-NP_Ce6_

**FIGURE 3:**
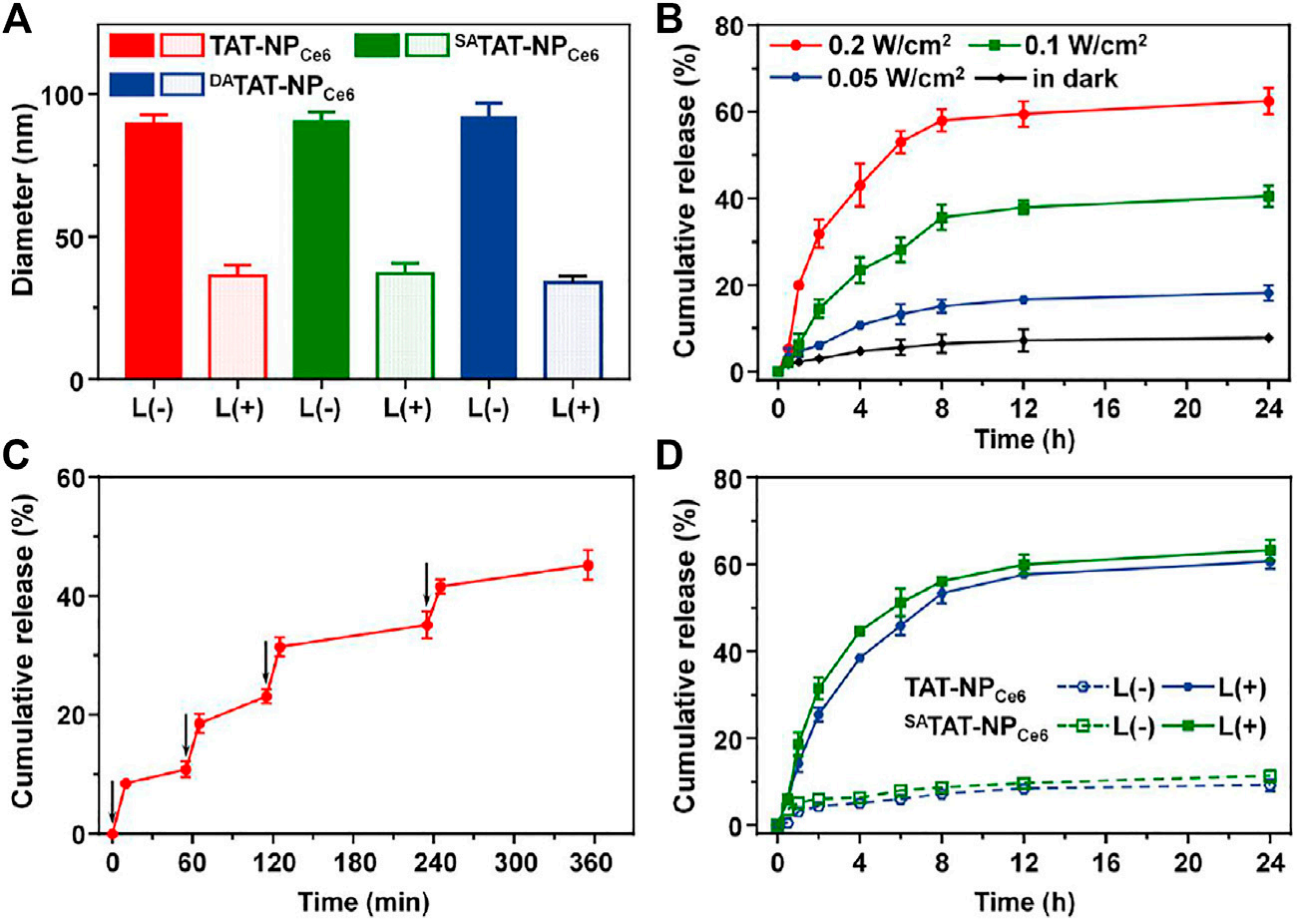
**(A)** Hydrodynamic size change of TAT-NP_Ce6_, ^SA^TAT-NP_Ce6_, and ^DA^TAT-NP_Ce6_ following 660 nm laser irradiation **(B)** The DOX release profile from ^DA^TAT-NP_Ce6_ under 660 nm laser irradiation. **(C)** Light-stimulated pulsed DOX release from ^DA^TAT-NP_Ce6_. The samples were irradiated with a 660 nm laser at different time points indicated by the arrows **(D)** The DOX release profile from TAT-NP_Ce6_ and ^SA^TAT-NP_Ce6_ under 660 nm laser irradiation (0.2 W/cm^2^).

According to our design, the generated ROS during the PDT process would selectively trigger the cleavage of TK linkers in nanocarriers to accelerate their disassembly and DOX release. We detected the thiol groups using Ellman’s test after exposure to a 660 nm laser (Supplementary Figure S2). The degradation of nanocarriers was obviously elevated with the extension of the irradiation times. There were 73.7, 72.9, and 71.3% of the TK linkages of TAT-NP_Ce6_, ^SA^TAT-NP_Ce6_, and ^DA^TAT-NP_Ce6_ cleaved after 660 nm laser irradiation for 60 min, respectively. On the contrary, their degradation in the dark was less than 5.0%. Next, the light-triggered disassembly of TK-bridged DOX-conjugated nanoparticles was measured through DLS. Following the irradiation of a 660 nm laser for 10 min, the size of the TAT-NP_Ce6_, ^SA^TAT-NP_Ce6_, and ^DA^TAT-NP_Ce6_ was obviously shrunk to ∼35 nm (Figure 3A). On the other hand, there was negligible size variation for all nanocarriers without laser exposure. The quantitative DOX release from ^DA^TAT-NP_Ce6_ under 660 nm laser exposure was further analyzed using HPLC. As shown in Figure 3B, the laser treatment at different power densities led to 18.13 ± 1.78, 40.51 ± 2.49, and 62.51 ± 3.05% of DOX release at 24 h, respectively. In contrast, only 7.76 ± 0.64% of total DOX was released from ^DA^TAT-NP_Ce6_ without laser treatment. After the exposure to various pulses of laser treatment, a controlled and pulsatile DOX release pattern was observed for ^DA^TAT-NP_Ce6_ (Figure 3C). More importantly, there was no significant difference in DOX release behavior of both non-responsive TAT-NP_Ce6_ and ^SA^TAT-NP_Ce6_ upon 660 nm laser irradiation (Figure 3D). Collectively, these results demonstrated that the external laser precisely triggered ^DA^TAT-NP_Ce6_ to disassembly and boosted drug release.

### Cellular Internalization at Different pH

**FIGURE 4:**
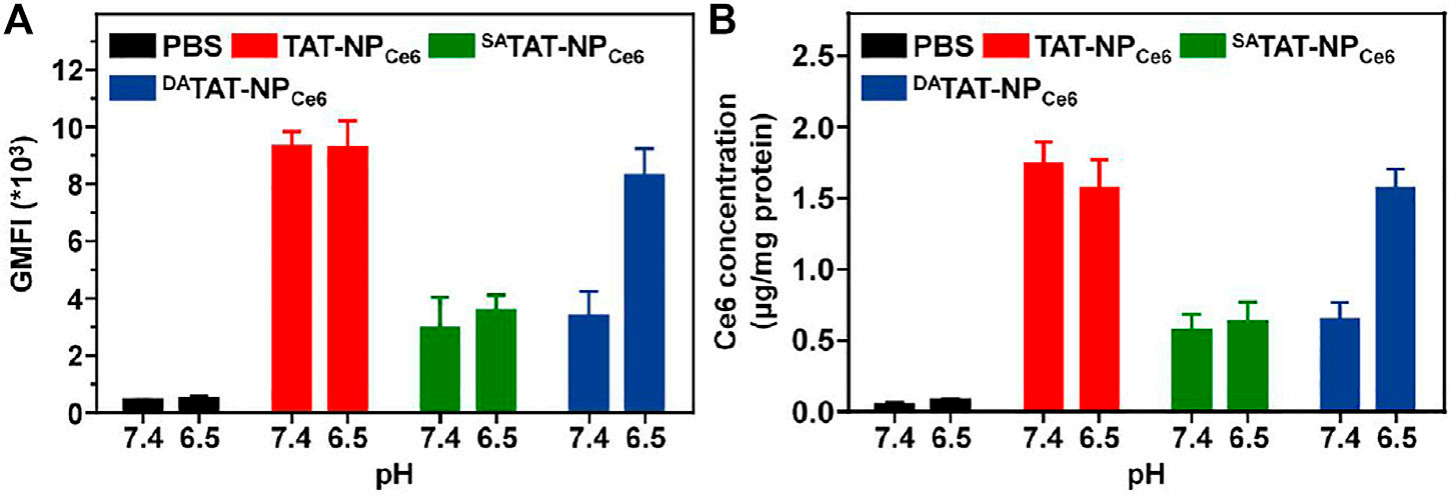
**(A)** The mean fluorescence intensity (MFI) of TAT-NP_Ce6_, ^SA^TAT-NP_Ce6_, or ^DA^TAT-NP_Ce6_ in MDA-MB-231 cells. **(B)** Quantitative analyses of Ce6 content in MDA-MB-231 cells [Ce6] = 6 μg/ml.

**FIGURE 5:**
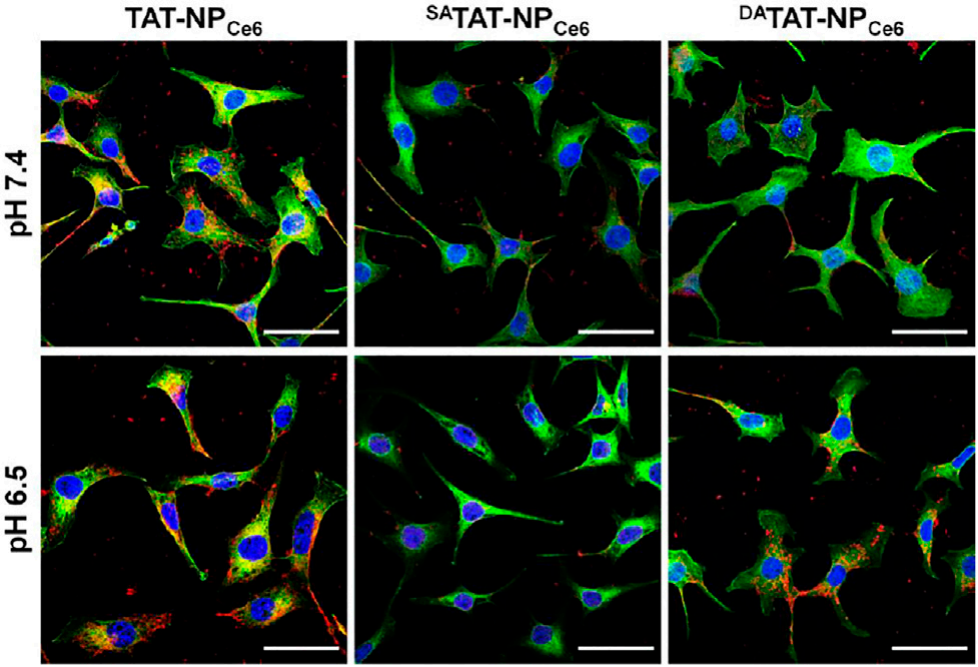
Cellular internalization of TAT-NP_Ce6_, ^SA^TAT-NP_Ce6_, and ^DA^TAT-NP_Ce6_ on MDA-MB-231 cells. DAPI and Alexa Fluor 488 phalloidin were used to stain cell nuclei and F-actin, respectively. The scale bar is 50 µm.

In order to evaluate the specific TAT presenting of ^DA^TAT-NP_Ce6_, the MDA-MB-231 cell line was chosen to investigate the cellular internalization. The various nanocarriers were pre-treated at either pH 7.4 or 6.5 for 2 h and then incubated with MDA-MB-231 cells for 2 h. The intracellular DOX fluorescence was analyzed by flow cytometry. As shown in Figure 4A, the mean fluorescence intensity (MFI) of MDA-MB-231 cells incubated with TAT-NP_Ce6_ was obviously the strongest among all groups at both pH conditions, suggesting that TAT moiety substantially facilitated the cellular uptake of the corresponding nanocarriers. Owing to the masking of TAT by non-responsive SA, the cellular uptake of ^SA^TAT-NP_Ce6_ was hindered at either pH 7.4 or 6.5. Notably, the intracellular DOX fluorescence of ^DA^TAT-NP_Ce6_ at pH 6.5 was significantly elevated compared to that of pH 7.4, suggesting the pH_e_-triggered DA degradation and TAT presenting. Meanwhile, the intracellular Ce6 content was quantitatively analyzed by HPLC after cell lysis, and the results (Figure 4B) further confirmed the increased cellular internalization of ^DA^TAT-NP_Ce6_ at pH 6.5. The intracellular Ce6 content at neutral pH of ^SA^TAT-NP_Ce6_ and ^DA^TAT-NP_Ce6_ group was 0.37- and 0.32-fold lower than that of the non-sensitive TAT-NP_Ce6_ group (1.74 ± 0.16 μg/mg protein), respectively. When the nanocarriers were pre-treated at acidic pH, the cellular internalization of ^DA^TAT-NP_Ce6_ was remarkably improved and increased to comparable to that of the TAT-NP_Ce6_ group, illustrating the pH_e_-mediated endocytosis of DA-masking nanoparticles. Furthermore, the promoted cellular internalization of ^DA^TAT-NP_Ce6_ at pH 6.5 was observed by a confocal laser scanning microscope. The filamentous actin and nuclei were stained by Alexa Fluor^®^ 488 Phalloidin and DAPI, respectively. In comparison with SA-masked ^SA^TAT-NP_Ce6_, a much stronger DOX signal was dominantly localized in the cytoplasm for cells treated with TAT-NP_Ce6_ and ^DA^TAT-NP_Ce6_ at pH 6.5 (Figure 5). These results confirmed the effectiveness of the pH_e_-induced TAT reactivable ^DA^TAT-NP_Ce6_, which provided a higher ability for specific tumoral cell targeting.

### Cytotoxicity *in Vitro*

**FIGURE 6:**
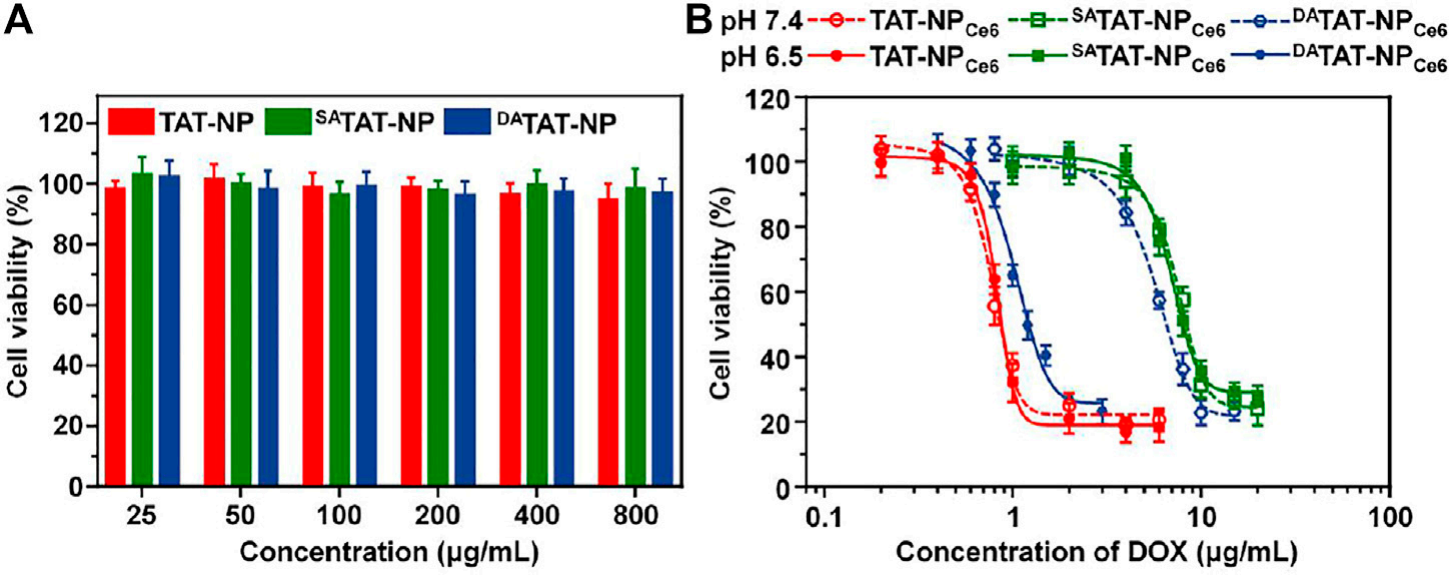
**(A)** Cytotoxicity of TAT-NP, ^SA^TAT-NP, and ^DA^TAT-NP on MDA-MB-231 cells for 72 h. **(B)** Combined chemo-PDT effect of TAT-NP_Ce6_, ^SA^TAT-NP_Ce6_, or ^DA^TAT-NP_Ce6_ on MDA-MB-231 cells under 660 nm laser irradiation.

As a combined chemo-PDT agent, the pH_e_-activated TAT presenting would further enhance the PDT efficacy of ^DA^TAT-NP_Ce6_ *via* increased PS internalization into tumoral cells. The ROS generated from Ce6 upon laser irradiation degraded TK linkers and initiated ^DA^TAT-NP_Ce6_ disassembly for subsequent DOX release. The cytotoxicity of various nanoparticles without cargo loading against MDA-MB-231 cells was first measured using the MTT assay, and there was negligible cytotoxicity at both pH conditions even when the concentration was 800 μg/ml (Figure 6A). Next, the cell viability after treatment with free Ce6 + DOX, TAT-NP_Ce6_, ^SA^TAT-NP_Ce6_, or ^DA^TAT-NP_Ce6_ was measured with 0.2 W/cm^2^ of 660 nm laser irradiation. As shown in Figure 6B, the cell viabilities were found to be dose-dependent in all the formulations. The significant difference between the TAT-NP_Ce6_+L and ^SA^TAT-NP_Ce6_+L group at either pH 7.4 or 6.5 can be attributed to the non-blocked cellular uptake *via* the TAT penetrating ligand. Since reactivable TAT has a weak acidity condition, the ^DA^TAT-NP_Ce6_+L group at pH 6.5 exhibited comparable cell killing to that of the TAT-NP_Ce6_+L group, with only 26.56 ± 2.17% of cell viability ([DOX] = 2.0 μg/ml). The IC_50_ value of the ^SA^TAT-NP_Ce6_+L group at pH 6.5 was 6.83 μg/ml, which was 8.33- and 6.62-fold higher than that of the TAT-NP_Ce6_+L and ^DA^TAT-NP_Ce6_+L groups, respectively. These results were consistent with the aforementioned experiments, demonstrating that the definitive efficiency of ^DA^TAT-NP_Ce6_+L at pH 6.5 is a result of both TAT-facilitated uptake and light-activated disassembly-induced DOX release.

### Pharmacokinetic and Biodistribution of ^DA^TAT-NP_Ce6_ *In Vivo*

**FIGURE 7:**
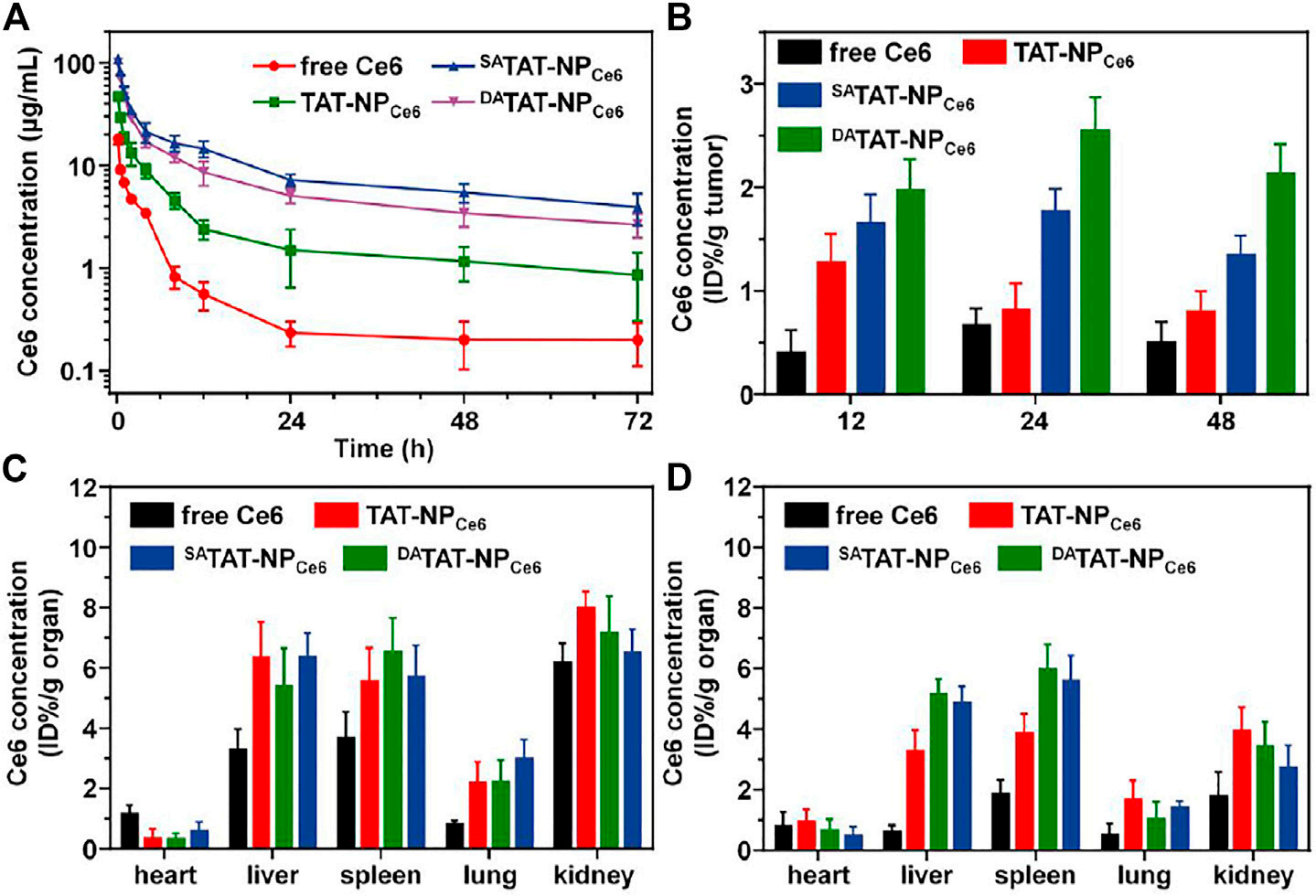
**(A)** Plasma Ce6 concentration *versus* time after systemic injection of free Ce6, TAT-NP_Ce6_, ^SA^TAT-NP_Ce6_, or ^DA^TAT-NP_Ce6_ (*n* = 4). **(B)** Quantitative Ce6 content in MDA-MB-231 tumors. Quantitative Ce6 content in major organs at 24 **(C)** and 48 h **(D)** post-injection.

As we expected, the TAT masking by either DA or SA would mask the TAT penetrating function and protect the nanocarrier from rapid clearance mediated by the TAT ligand. We analyzed the blood circulation of all formulations in BLAB/c mice following *i. v.* injection. Compared to TAT-NP_Ce6_, which was cleared from the bloodstream in the first 12 h, both DA-masking nanocarriers (^SA^TAT-NP_Ce6_ and ^DA^TAT-NP_Ce6_) noticeably increased Ce6 retention even at 72 h post-injection (Figure 7A). The Ce6 concentration of TAT-NP_Ce6_, ^SA^TAT-NP_Ce6_, and ^DA^TAT-NP_Ce6_ at 72 h post-injection was 0.86 ± 0.56, 3.94 ± 1.35, and 2.66 ± 0.69 μg/ml, respectively. The obviously decreased Ce6 concentration in plasma of -TAT-NP_Ce6_ at 72 h post-injection compared to ^SA^TAT-NP_Ce6_ was mainly attributed to a small amount of DA degradation at neutral pH, which is in line with previous reports (Li et al., 2017; Ma and Sun, 2020). Based on the non-compartmental model, the TAT-masking strategy prolonged the area under the curve (AUC_0-t_) of ^SA^TAT-NP_Ce6_ and ^DA^TAT-NP_Ce6_ to be 715.78 ± 48.40 and 515.28 ± 25.50 μg/ml*h, respectively.

The improved pharmacokinetic profiles offer more opportunities for ^SA^TAT-NP_Ce6_ and ^DA^TAT-NP_Ce6_ to enrich tumor tissues *via* the EPR effect. We next quantitatively studied the biodistribution of ^DA^TAT-NP_Ce6_. The MDA-MB-231 tumor-bearing BALB/c nude mice were treated with nanocarriers through the tail vein and sacrificed at 12, 24, and 48 h. The Ce6 concentration in major organs and tumor tissues was detected by HPLC. Although both ^SA^TAT-NP_Ce6_ and ^DA^TAT-NP_Ce6_ showed similar extravasation to the tumor matrix *via* the EPR effect, the DA degradation and subsequent TAT regeneration significantly facilitated the tumor accumulation by boosting tumoral cell internalization. The Ce6 content of ^DA^TAT-NP_Ce6_ group was 1.97 ± 0.30, 2.55 ± 0.32, and 2.13 ± 0.28% ID/g tumor at 12, 24, and 48 h post-injection, respectively (Figure 7B). On the contrary, TAT-NP_Ce6_ and ^SA^TAT-NP_Ce6_ groups only reached up to 0.80 ± 0.20 and 1.35% ± 0.18 ID/g tumor at 48 h post-injection, respectively. Meanwhile, all nanoparticular formulations realized elevated Ce6 concentration in reticuloendothelial system (RES) organs (e.g., liver and spleen, Figure 7C&7D), which is in agreement with the previous literature (Yeo et al., 2018; He et al., 2020).

### Antitumor Effect *in Vivo*

**FIGURE 8:**
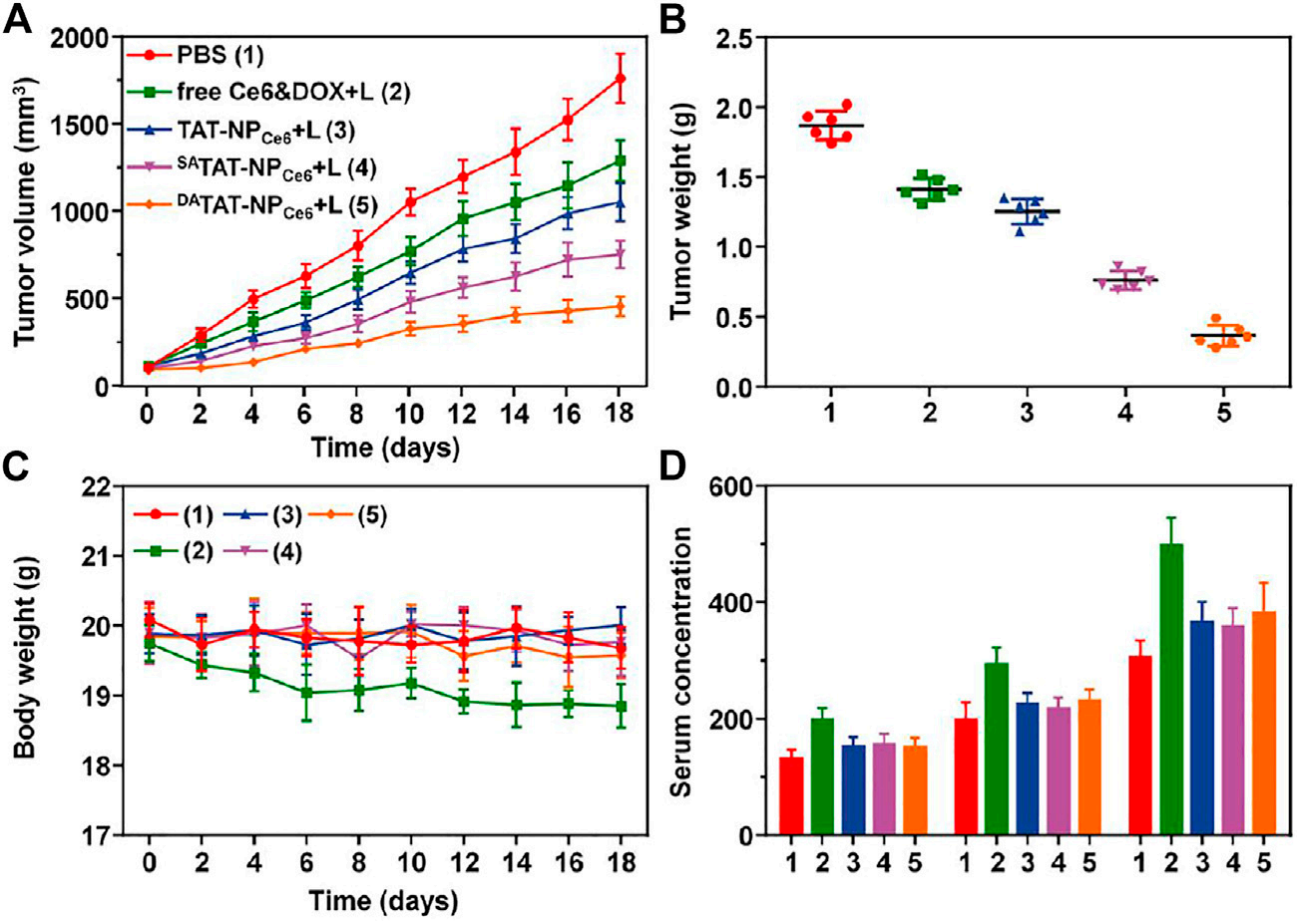
**(A)** Tumor volume in MDA-MB-231 tumor xenograft-bearing nude mice received various treatments. The *i.v.* injections were performed on days 0, 7, and 14 (*n* = 6). **(B)** The MDA-MB-231 tumor mass 1 day after the treatment (day 19). **(C)** Body weight of the mice was monitored on different days. **(D)** ELISA examination of ALT (U/L), AST (U/L), and BUN (10 nmol/ml) in the serum on day 19.

Inspired by the prolonged blood circulation and preferential tumor accumulation, we next investigated the antitumor effect of ^DA^TAT-NP_Ce6_ *in vivo*. Thirty MDA-MB-231 tumor-bearing mice were divided into five groups and received a systemic injection of PBS, free Ce6&DOX, TAT-NP_Ce6_, ^SA^TAT-NP_Ce6,_ or ^DA^TAT-NP_Ce6_ at an equivalent DOX dose of 5.0 mg per kg body weight every week, respectively. At 24 h post-injection, the tumor tissues were irradiated with a 660 nm laser (200 mW/cm^2^) for 10 min. As illustrated in Figure 8A, the tumor volume of the PBS control group sharply reached approximately 1763.9 mm^3^ on day 18. Due to the DOX being responsively liberated and chemotherapy initiated, the tumor growth in both TAT-NP_Ce6_ and ^SA^TAT-NP_Ce6_ groups was partially inhibited through a combined chemo-PDT process, and their difference could be attributed to the increased tumor enrichment *via* SA-masking TAT peptide. In contrast, the ^DA^TAT-NP_Ce6_ plus laser irradiation showed the most remarkable anticancer efficiency, and the tumor inhibition rate was only up to 74.25% on the last day. After the sacrifice of mice on day 19, the smallest tumor weight of the ^DA^TAT-NP_Ce6_+L group (0.37 ± 0.08 g) further verified its advanced anticancer effect (Figure 8B). On the other hand, in comparison with the free Ce6&DOX + L group whereas the body weight substantially declined, the body weight of mice treated with various nanocarriers remained normal during the whole therapeutic window (Figure 8C). According to the ELISA results of ALT, AST, and BUN, liver and kidney damage is believed to be negligible after the ^DA^TAT-NP_Ce6_+L treatment (Figure 8D). Additionally, blood routine count evaluation in Supplementary Table S1 further demonstrated the biosafety and biocompatibility of ^DA^TAT-NP_Ce6_ *in vivo*.

Although CPPs can promote nanocarriers into tumoral cells more efficiently, the rapid degradation in blood circulation and non-specific targeting for normal organs hinder their applications. In comparison with the strategy to expose CPPs by removing stealth polymer (Koren et al., 2012; Jing et al., 2022), CPP masking by specific moieties does not rely on steric effect but fundamentally shields the CPP function to achieve more stable blood stealth. More importantly, H^+^-sensitive TAT presenting of ^DA^TAT-NP_Ce6_ is much faster than previous systems respond to other extracellular stimuli (e.g., enzyme and laser) (Mo and Gu., 2016; Du et al., 2018; Zhu et al., 2013; Zhang et al., 2022). In this study, the characterizations for DA degradation and other experiments demonstrated that ^DA^TAT-NP_Ce6_ realized more efficient TAT activation and specific tumoral cell internalization. Except for cellular uptake, efficient payload release is necessary for nanocarriers because most therapeutic agents function intracellularly. The light-triggered DOX release of ^DA^TAT-NP_Ce6_ achieved cascaded chemo-PDT for breast cancer. Compared to the previous studies which focused on CPPs presenting and light-controlled cargo release alone (Jiang et al., 2018; Xu et al., 2021; Zhang et al., 2021), we integrated both functions in ^DA^TAT-NP_Ce6_ through a facile mixed micelle method. More interestingly, the ratio of TAT-PEG-PHEP and PEG-PPE-DOX, therapeutic agent, and photosensitizer could be easily adjusted during micelle preparation to regulate the performance of ^DA^TAT-NP_Ce6_ (e.g., targeting ability and drug loading content) for more specific and precise treatment of various cancers in the future. This work provides a new point of view to prepare a hieratical-activable nanocarrier for combined chemo-PDT. Further integration of multimodality diagnosis function (e.g., MR, CT, or photoacoustic imaging) is encouraged through a similar approach for promising theranostics.

## Conclusion

In summary, we have proposed a bioresponsive nanocarrier for tumor-specific drug delivery and “on-demand” chemo-PDT therapy for breast cancer. Through the TAT-masking strategy, ^DA^TAT-NP_Ce6_ avoided unexpected clearance in the bloodstream and specifically realized advanced tumor accumulation *via* pH_e_-triggered DA degradation and TA function regeneration. Upon 660 nm red laser irradiation, the PDT effect of Ce6 not only directly killed the tumoral cells but also boosted the cascaded chemotherapy through ROS-induced TK bond breakage and micelle disassembly. Considering the specific tumor acidity and controllability of laser in spatial, the cell-killing effect precisely occurred within tumor sites. This study provides an attractive strategy for fabricating stimuli-responsive nanoparticles for combined chemo-PDT therapy.

## Data Availability

The raw data supporting the conclusion of this article will be made available by the authors, without undue reservation.
