# Supplementary material for: Tumor pH-Responsive Nanocarriers With Light-Activatable Drug Release for Chemo-Photodynamic Therapy of Breast Cancer
Source: Front Chem. 2022 Jun 22;10:905645. doi: 10.3389/fchem.2022.905645 (PMC9257215; doi:10.3389/fchem.2022.905645)
Supplement: Supplementary file 1 [file DataSheet1.docx]

Supplementary Material


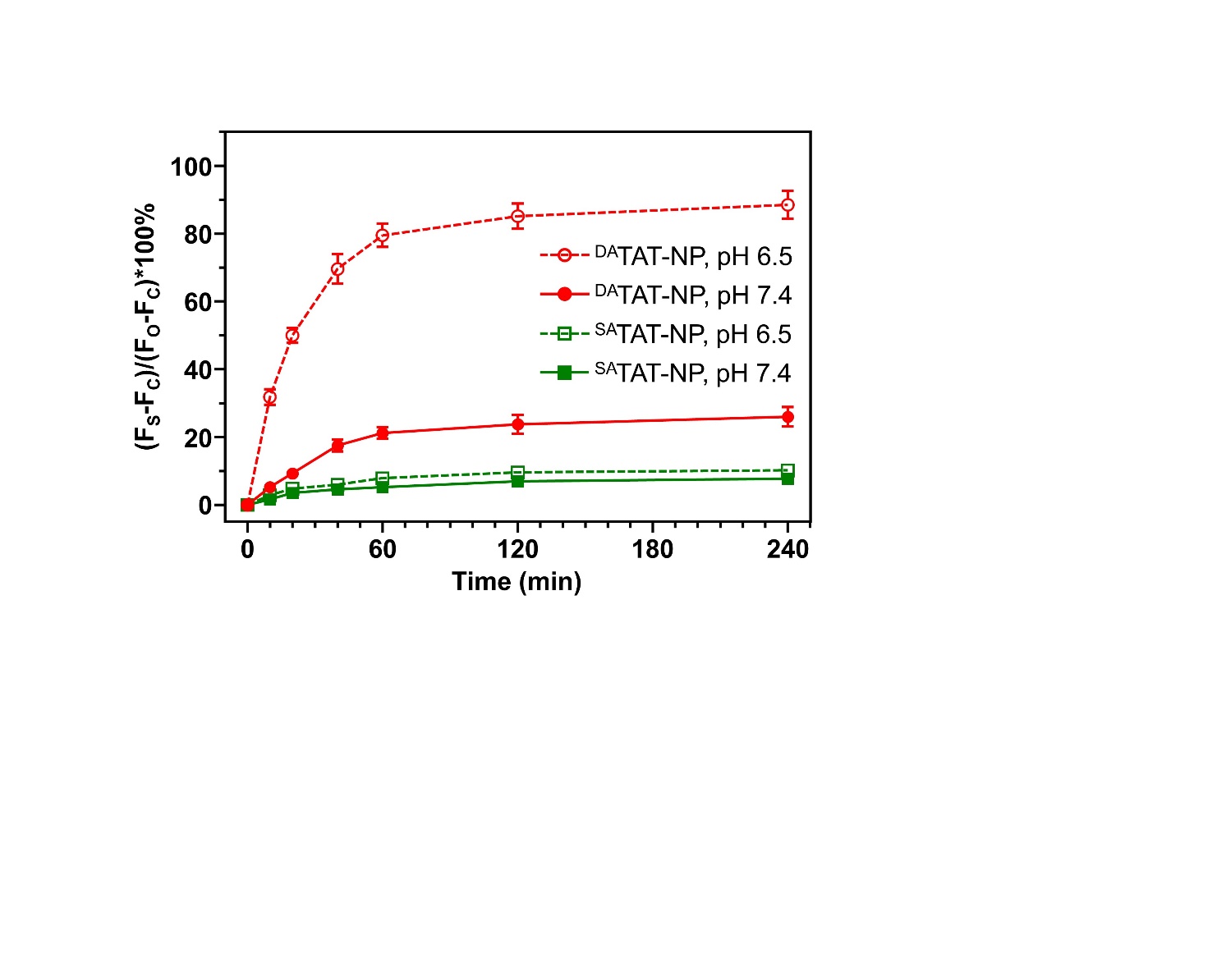


**Figure S1.** Degradation of nanocarriers using fluorescamine as the sensor. The fluorescence intensity (Fs) of ^SA^TAT-NP or ^DA^TAT-NP was detected (E_x_: 390 nm, E_m_: 483 nm). F_O_ was defined as the fluorescence of TAT-NP at same concentration. F_C_ was defined as the fluorescence of PBS control.


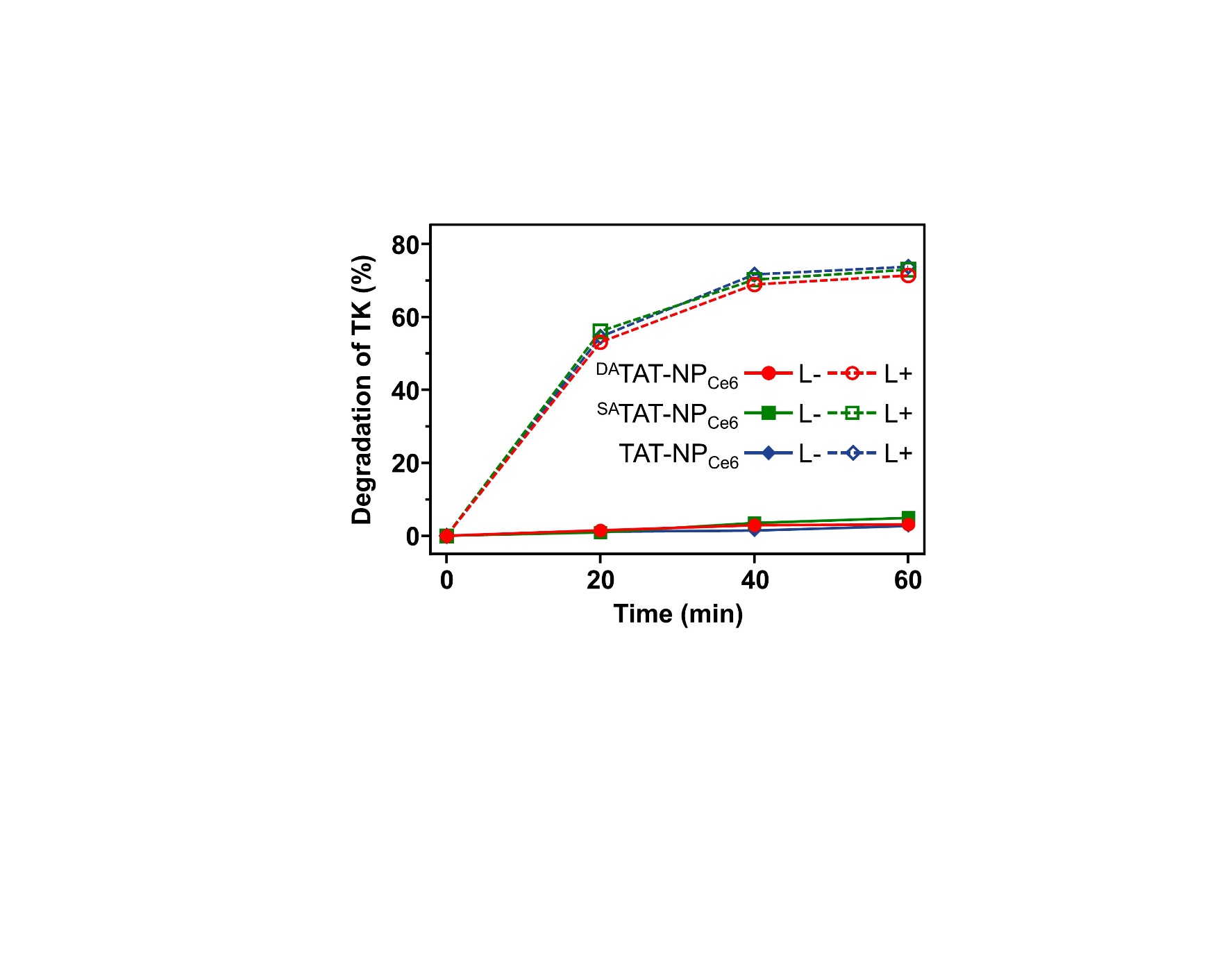


**Figure S2.** TK linker degradation of TAT-NP_Ce6_, ^SA^TAT-NP_Ce6_ and ^DA^TAT-NP_Ce6_ with or without 660 nm laser irradiation.

|  | PBS | TAT-NP_Ce6_ | ^SA^TAT-NP_Ce6_ | ^DA^TAT-NP_Ce6_ |
| --- | --- | --- | --- | --- |
| WBC | 4.56±0.27 | 4.67±0.33 | 4.49±0.28 | 4.34±0.13 |
| RBC | 10.52±0.18 | 10.35±0.27 | 10.69±0.35 | 10.73±0.30 |
| HGB | 165.17±7.94 | 169.50±7.31 | 167.33±4.41 | 165.83±5.78 |
| HCT | 36.42±3.72 | 34.47±3.65 | 35.22±3.07 | 34.23±3.14 |
| MCV | 35.13±3.17 | 35.60±3.32 | 32.95±2.05 | 36.88±2.99 |
| MCH | 17.35±0.89 | 16.65±0.69 | 17.50±0.96 | 17.45±0.79 |

**Supplementary Table 1.** Routine blood analysis of BALB/c mice treated with different formulations. WBC: white blood cells, RBC: red blood cells, HGB: hemoglobin, HCT: hematocrit, MCV: mean corpuscular volume, MCH: mean corpuscular hemoglobin.
